# Using a Safe System Framework to Examine the Roadway Mortality Increase Pre-COVID-19 and in the COVID-19 Era in New York State

**DOI:** 10.3390/ijerph22010061

**Published:** 2025-01-03

**Authors:** Joyce C. Pressley, Zarah Aziz, Emilia Pawlowski, Leah Hines, Aisha Roberts, Jancarlos Guzman, Michael Bauer

**Affiliations:** 1 Department of Epidemiology and Health Policy and Management and the Columbia Center for Injury Science and Prevention, Columbia University, New York, NY 10032, USA; 2 Department of Epidemiology, Columbia University, New York, NY 10032, USA; zarahfaziz@gmail.com; 3 New York State, Bureau of Occupational Health and Injury Prevention, Albany, NY 12237, USA; emilia.pawlowski@health.ny.gov (E.P.); leah.hines@health.ny.gov (L.H.); aisharoberts1103@gmail.com (A.R.); jancarlos.guzman@health.ny.gov (J.G.); michael.bauer@health.ny.gov (M.B.)

**Keywords:** motor vehicle crash, mortality, safe system, COVID-19

## Abstract

Roadway mortality increased during COVID-19, reversing a multi-decade downward trend. The Fatality Analysis Reporting System (FARS) was used to examine contributing factors pre-COVID-19 and in the COVID-19 era using the five pillars of the Safe System framework: (1) road users; (2) vehicles; (3) roadways; (4) speed; and (5) post-crash care. Two study time periods were matched to control for seasonality differences pre-COVID-19 (*n* = 1725, 1 April 2018–31 December 2019) and in the COVID-19 era (*n* = 2010, 1 April 2020–31 December 2021) with a three-month buffer period between the two time frames excluded. Four of the five pillars of the safe system had road safety indicators that worsened during the pandemic. Mortality was 19.7% higher for motor vehicle occupants and 45.1% higher for riders of motorized two-wheeled vehicles. In adjusted analyses, failure to use safety equipment (safety belts/helmets) was associated with 44% higher mortality. Two road user groups, non-motorized bicyclists and pedestrians, did not contribute significantly to higher mortality. Urban roadway crashes were higher compared to rural crashes. Additional scientific inquiry into factors associated with COVID-19-era mortality using the Safe System framework yielded important scientific insights to inform prevention efforts. Motorized two-wheeled vehicles contribute disproportionately to pandemic-era higher mortality and constitute an emerging road safety issue that deserves further attention.

## 1. Introduction

The arrival of COVID-19 was accompanied by a reversal of the downward trend in motor vehicle mortality that initially caught road scholars by surprise [1]. In the year prior to COVID-19’s appearance, New York State experienced a 3.4% decrease in motor vehicle mortality [2]. Stay-at-home orders; closures of restaurants, bars and entertainment venues; movement to work-from-home; a rise in internet shopping; and a move to virtual education lowered vehicle miles traveled and left city streets, county roads, and sidewalks with fewer travelers [3,4,5,6]. While the scope and magnitude of the mortality changes vary, there is remarkable consistency in the sustained increase across the majority of U.S. states [7,8,9,10,11,12].

While several road user behavioral factors have been implicated, other pillars of the Safe System framework have been neglected with regard to scientific inquiry into their potential contributions. The Safe System’s five pillars are objectives to support Safe System principles that ‘serious road injury is unacceptable, that humans are vulnerable and make mistakes, responsibility is shared, safety is proactive and redundancy is crucial’ [13,14,15]. Previous reports of motor vehicle mortality lend support to our hypotheses that other pillars of the Safe System framework, in addition to road user behavior, deserve further scientific inquiry [16,17,18].

This study compares pre-COVID-19 and COVID-19-era fatal crashes across the five pillars of the Safe System: (1) road users; (2) vehicles; (3) roadways; (4) speed; and (5) post-crash care. Two time periods are selected for comparison that are matched to control for seasonality differences across the pre-COVID-19 and COVID-19-era periods. Additional scientific inquiry into factors associated with higher COVID-19-era mortality could yield important scientific insights to inform future prevention efforts [19,20,21,22].

This paper is an important contribution to the literature as it uses a large sample size, an extended timeframe seasonally and geographically matched with a pre-pandemic control population, and a well-defined injury outcome (death) to explore factors hypothesized to contribute to this continued increase in mortality. Understanding these factors is paramount to returning our roadway mortality to its pre-pandemic trajectory.

## 2. Materials and Methods

The Fatality Analysis Reporting System (FARS) [23,24,25] is used to investigate factors hypothesized to be associated with the higher pandemic-era motor vehicle mortality in New York State.

### 2.1. Study Population

The study population included 3735 deaths occurring on a public roadway during two study time frames. A baseline pre-COVID-19 era study included 1725 fatal crash deaths occurring between 1 April 2018 and 31 December 2019. The COVID-19 era included 2010 fatal crash deaths occurring between 1 April 2020 and 31 December 2021. A buffer period from 1 January 2020 to 31 March 2020 was excluded when COVID-19 was beginning [26,27]. The study population included all road user types captured by FARS [23].

### 2.2. Data Source(s)

FARS is made available by the National Highway Traffic Safety Administration as a deidentified public use data set [23,24]. It contains a census of all fatal crashes on a U.S. roadway and provides information on the person type (driver, passenger, or pedestrian), vehicle type and crash characteristics, roadway features, and post-crash transport. All New York State roadway deaths captured by FARS during the two identified study periods were analyzed.

### 2.3. Variable Classifications

Key FARS variables were mapped to each of the five pillars of the Safe System framework [16,17,18].

*Outcome variable.* Mortality that occurred on a public roadway in New York State was compared for the two study periods using binominal multivariable logistic regression.

*Covariates.* FARS variables mapped across the five pillars of the Safe System framework comprised the independent covariates of the binominal logistic regression model.

### 2.4. Pillar 1: Road User-Level Characteristics

#### 2.4.1. Demographics and Person Type

*Driver ages* were categorized as shown in Table 1. For descriptive purposes, all motor vehicle occupants were characterized as: child (0–12), teen (13–19), younger adult (20–44), middle-aged adult (45–64), and older adult (65 and older). Sex was captured in FARS as a dichotomous variable.

*Person type*. Fatalities were categorized as: (1) driver or passenger of a four-wheeled motor vehicle; (2) a motorcycle, moped or other motorized two- and three-wheeled vehicles; (3) bicycle, not motorized; (4) pedestrian; or (5) other/unknown.

#### 2.4.2. Driver/Occupant Behavior

##### Use of Protective Gear

*Occupant restraint* was categorized as a dichotomous variable, restrained or not restrained, with use of any type of restraint categorized as restrained.

*Helmet use*. For motorized two- and three-wheeled vehicles, the use of a helmet was classified as present or absent. Helmet wearing was not available for non-motorized bicyclists.

*Driver license*. License was considered valid if the driver had a valid learner’s permit, intermediate or full license, or a temporary license. Invalid included no license or a revoked, expired, suspended, or canceled license.

*History of risky driving behavior*. Risky driving behavior was categorized as several dichotomous variables (Table S1) and as a composite variable consisting of a count of the total and of the types of risky driving behaviors within the last three years (Table 1). Drivers were classified as having a risky driving history if, within the three years prior to the fatal crash, their record demonstrated a previous citation for (1) driving under the influence/driving impaired; (2) speeding; (3) driving with a suspended, revoked, canceled, or expired driver license; or another moving violation.

*Driver drug or alcohol status.* This variable was categorized as negative, positive, or not tested. A positive status included any of the following: (1) police reported alcohol involvement; (2) driver blood alcohol concentration over the legal limit; or (3) another positive drug or alcohol test.

### 2.5. Pillar 2: Vehicle and Vehicle Crash Level Characteristics

*Vehicle model type.* Vehicle type was categorized as passenger car, utility vehicle (SUV), van, small or large pickup truck, motorized two-wheeled vehicle and other. Vehicles such as large trucks, farm equipment, and buses were classified as “other”.

*Motorized two- or three-wheeled vehicle*. This variable was examined as a total and by specific type of vehicle, including two-wheeled motorcycles (*n* = 621), three-wheeled motorcycles (*n* = 8), off-road motorcycles (*n* = 18), and moped/motor scooter/minibikes (*n* = 51).

*Single or multiple vehicle collisions*. Collisions were characterized as single (one vehicle), two vehicles, or more than two vehicles.

*Collision type*. Collision types were categorized as head-on, angle, sideswipe, rear-end, or other. Non-collision was defined as crashes that did not involve collision with a motor vehicle in transport.

*Vehicle maneuver at time of crash*. Vehicle action at the time of crash was collapsed due to small cell sizes: (1) going straight; (2) turning (left, right or U); (3) negotiating a curve; (4) passing or overtaking; and (5) other/unknown.

*Vehicle rollover and ejection*. Rollover was classified as dichotomous (rollover and no rollover). Ejected was collapsed into (1) not ejected and (2) ejected, partial or full.

### 2.6. Pillar 3: Roadway Characteristics

*New York State Regions.* Three regions of New York State were examined. New York City comprises five boroughs (counties): New York, Bronx, Queens, Kings, and Richmond (Staten Island). Long Island comprises the Suffolk and Nassau counties. Upstate comprises the 55 remaining counties [28].

*Roadway features.* Several roadway characteristics were examined including urban/rural roadway, number of lanes, divided roadway, intersection, roadway surface, weather, traffic control devices, and lighting conditions.

### 2.7. Pillar 4: Speeding

Speed was assessed using two variables: speed-related crash and history of ticketing for speeding in the last 3 years. Actual miles per hour traveled was not available for analysis [23].

### 2.8. Pillar 5: Post Crash Care

Post crash care was examined for the following: (1) died at the crash scene, not transported; (2) died en route; and (3) alive at emergency department arrival. Times examined were from crash to emergency medical service arrival and time from crash to hospital arrival. Persons who died at the scene were excluded from crash time to hospital arrival. *Time crash occurred*. This variable was used in conjunction with two other variables as the basis for estimating time from crash to emergency medical service arrival (out-of-hospital care) and time from crash to hospital arrival. All calculated time variables are in minutes. *Time from crash to emergency medical service arrival at the scene (minutes)*. This constructed variable is a calculation of the difference between the time of crash occurrence and time of emergency medical service arrival on the scene. This variable is reported for the total population emergency medical service responses regardless of whether they were transported and is also stratified by: (1) died at the crash scene; (2) died en route to the hospital; and (3) did not die in route or at the scene. *Time from crash to arrival at hospital (minutes).* This constructed variable is calculated for transported crash victims from the time of crash occurrence to the time of emergency medical service arrival at the hospital. Persons who died en route are included, but persons considered dead on arrival at the scene were excluded from crash time to hospital arrival calculations.

### 2.9. Statistical Analysis

FARS data variables were mapped to the five pillars of the Safe System framework. Two time periods were constructed from these four years of data that allowed us to control seasonality by matching the month of fatal crash in the two time periods. The pre-COVID period included 1 April 2018–31 December 2019 and the COVID-19 era was 1 April 2020–31 December 2021. A transition period of 3 months (1 January 2020 to 31 March 2020) was excluded as a transition period that included the time frame when COVID-19 was beginning to emerge as an infectious disease. April 2020 was the first full month of data after the COVID-19-related restaurant and entertainment venues had been shuttered in New York State and comprised the beginning of Time Period 2, the COVID-19 era. Descriptive statistics were examined for variables of interest representing each of the five pillars. All data were examined for numerical characteristics using bivariable analyses before being used in multivariable models. Chi square, logistic regression and multi-level logistic regression were used to analyze data [29]. Our goal was to investigate characteristics of fatal crashes across the two time periods immediately before COVID-19 and compared to the COVID-19 era. To examine differences in mortality risk factors between the Pre-COVID-19 and the COVID-19 eras, unadjusted and adjusted binominal logistic regression models were constructed with mortality in the two periods as the outcome variable and variables mapped across the five pillars as the independent covariates. All statistical analyses were two-sided and a *p*-value < 0.05 was considered statistically significant. All analyses were performed in R [30,31].

## 3. Results

**Figure 1:**
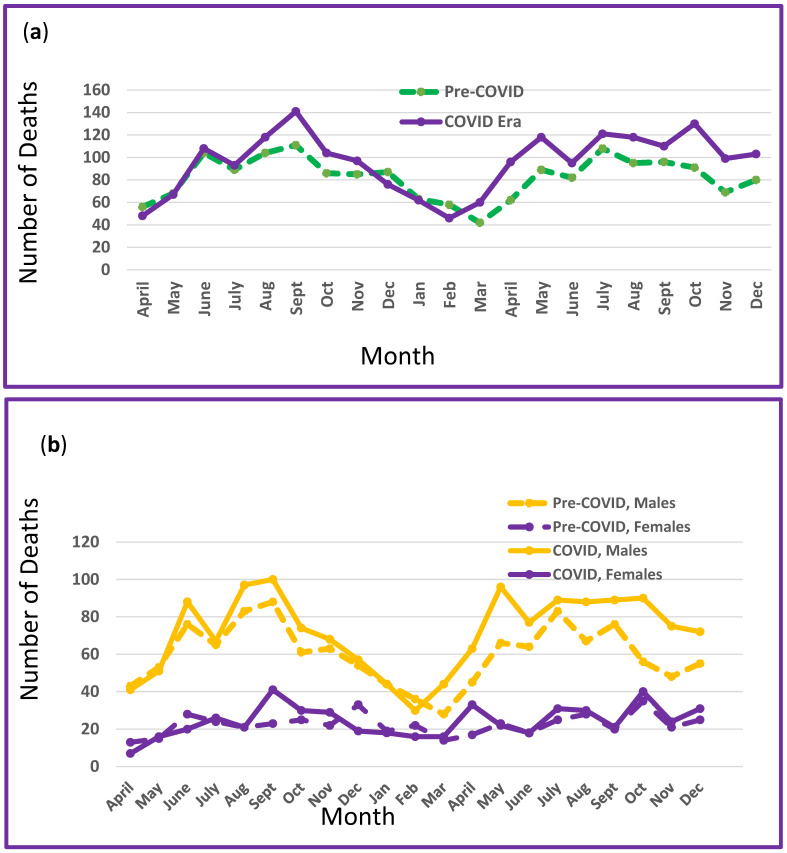
(**a**) Total mortality on New York State roadways: pre-COVID-19 (dashed lines) and in the COVID-19 era (solid lines). (**b**) Mortality on New York State roadways by sex: pre-COVID-19 (dashed lines) and in the COVID-19 era (solid lines).

New York State experienced a total of 3735 fatalities during the 42 months comprising the two periods of this pre-COVID-19 and COVID-19 era study (Table 1). Total mortality was 16.5% higher during the COVID-19 era compared to the pre-COVID-19 baseline time frame with the proportion of increase differing significantly by crash category and person type. Motor vehicle occupants accounted for approximately half of all fatalities and pedestrian injuries for one-quarter. The remaining deaths were from motorized two- and three-wheeled vehicles (19%) and non-motorized bicycles (4%). Total mortality and mortality by sex are shown by month for the two time periods, as shown in Figure 1a,b. Males exhibited higher mortality and greater seasonal variation than females, but sex differences were not significantly different between the two study periods.

### 3.1. Pillar 1: Road User Characteristics

#### 3.1.1. Road User Type

Motor vehicle occupant mortality was 19.7% higher with driver mortality higher by 18.0% and accounting for nearly 76% of all occupant mortality. Two road user groups, non-motorized bicyclists, and pedestrians, accounted for an insignificant proportion of the higher pandemic-associated mortality. Motorcycles and motorized two-wheeled vehicle mortality was 45.1% higher. This was primarily due to drivers of motorcycles/motorized two-wheeled vehicles, who had 46.5% higher mortality, while passenger mortality for these vehicles was 20.0% higher in the pandemic era. Non-motorized bicycle mortality showed a smaller mortality change during the COVID-19 era (4.2%) while pedestrian deaths were 6.6% lower during the pandemic. Passengers of motor vehicles accounted for a smaller proportion of occupants and had 25.0% more deaths during the pandemic.

Although pandemic mortality associated with having a history of several risky driving behaviors in the COVID-19 era tended to be higher across several categories (Table S1), only history of prior speeding violation (66.7% higher) was statistically significant (Table 1).

#### 3.1.2. Motor Vehicle Occupants

There were significant differences in age between the two study periods with more mortality occurring in drivers aged 30–34 and 35–39 years (Table 1). Drivers aged 45 years and older exhibited comparatively small increases while mortality in drivers aged 65 years and over declined slightly. The proportion of drivers who died unrestrained during the pandemic was 40% higher compared to a 5.7% higher mortality in those who were restrained (Table 1).

#### 3.1.3. Motorized Two- and Three-Wheeled Vehicles

Mortality in motorized two- and three-wheeled vehicles comprised 18.8% of total traffic mortality in this study population, with 95% of those deaths being drivers of the vehicles (Table 1). Overall, motorized two- and three-wheeled vehicle mortality increased by 45% during the COVID-19 era. The proportion of deaths involving helmetless riders increased from 6.3% pre-COVID-19 to 14.4% during the pandemic. While mortality in helmeted riders increased by only 35%, the increase was 235% in the helmetless (Table 1).

#### 3.1.4. Pedestrians

Overall pedestrian deaths declined during the COVID-19 era, but this decrease was concentrated in the early COVID era (Figure S1) and was uneven across the age span (Table 1). The overall decline was driven by a more than 20% drop in the 65 years and older age group (Table 1). Males comprised 60% of pedestrian deaths in both study periods. Mortality declines were seen for both males and females, but the sharpest decline was observed for males (9.0% vs. 2.9%). Two groups, ages 45–64 and 65 and over comprised nearly two-thirds of all pedestrian deaths and experienced a 7.9% and 22.7% decline, respectively.

### 3.2. Pillar 2: Vehicle Characteristics

There were significant differences in the types of vehicles involved in fatal crashes between the two study periods (Table 2). In addition to the increase in motorcycle deaths, the most notable change was the increase in roadway deaths involving motorized mopeds, scooters, minibikes, and off-road motorcycles. The absolute number of deaths involving these vehicles increased between the two periods (*n* = 15 to 54, *p* < 0.001) with a percent change between the two periods of more than 200%. Mortality associated with other off-road vehicles, such as ATVs and UTVs, was 42.3% higher. Smaller increases were observed for cars, SUVs, vans, and pickup trucks (Table 2).

Vehicle passing or overtaking accounted for approximately 3% of pre-COVID-19 vehicle crashes involving a fatality, but nearly doubled during the COVID-19 era (Table S2). The largest percent change in vehicle collisions involved multi-vehicle crashes with more than two vehicles (56.2%) (Table 2). Two-vehicle collisions increased by more than 20% while single-vehicle collisions showed the smallest increase. Vehicle sideswipes and rear end collisions contributed significantly to the increased mortality (Table 2).

### 3.3. Pillar 3: Roadway Characteristics

Several roadway characteristics were associated with an increase in fatalities during the COVID-19 era.

#### Urbanization

**Figure 2:**
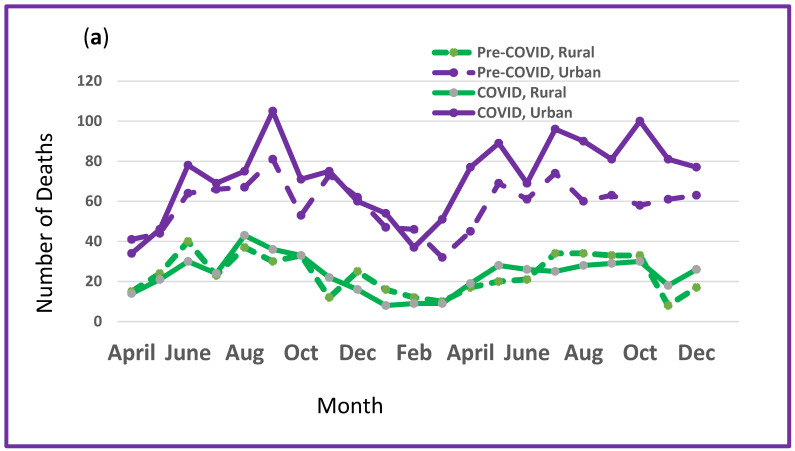

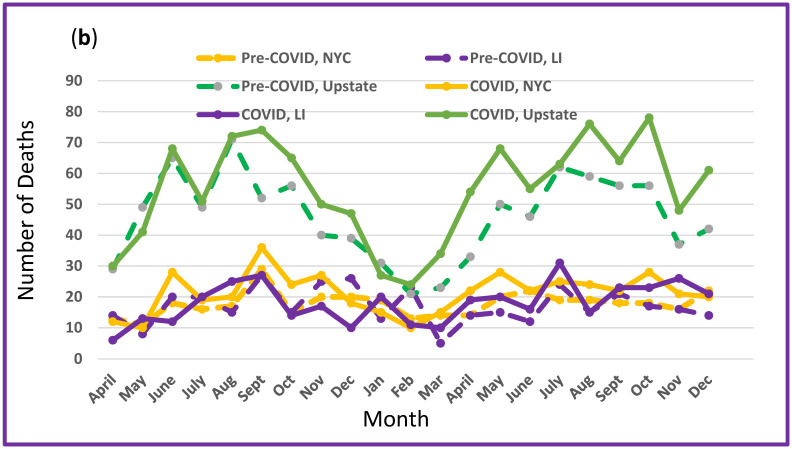
(**a**) Mortality on New York State roadways for urban (purple) and rural (green) areas: pre-COVID-19 (dashed lines) and in the COVID-19 era (solid lines). (**b**) Total mortality by month for Upstate, Long Island and New York City: pre-COVID-19 (dashed lines) and in the COVID-19 era (solid lines).

Mortality increased more on urban than rural roadways. More than 70% of fatalities occurred on urban roadways, which saw a 23.2% increase in fatalities during the pandemic (Table 2) (Figure 2a).

All regions of New York State experienced an increase in road mortality during the COVID-19 era, but the percent increase was highest in Upstate and New York City, with Long Island experiencing smaller increases (Table 2) (Figure 2b). Upstate experienced greater seasonal variation in mortality during both study periods than did either New York City or Long Island.

Four-way intersections had a very modest increase (1.9%) while Y and T intersections had a significant decline. Fatalities categorized as not at an intersection (*n* = 2500) had the largest increase in the absolute percentage of fatalities (61.2% to 70.9%). Fatal crashes associated with driving in inclement weather were lower during the pandemic (Table 2).

### 3.4. Pillar 4: Speeding

The only speed-related variable associated with a significant change in mortality during the pandemic was having a history of being ticketed during the last three years for speeding (Table 1). Speeding at the time of the fatal crash increased from 32.7% of fatalities pre-COVID-19 to 36.4% during the COVID-19 era (Table 2). Speed-related crashes exhibited a 31.3% higher mortality compared to a 11.7% higher mortality in crashes not noted to be speed-related.

### 3.5. Pillar 5: Post Crash Care

#### 3.5.1. Mode of Transport

Post crash transport differed significantly with the COVID-19 era being associated with a 30% higher proportion who were not transported and a 9.4% higher proportion transported by ground ambulance (Table 2). There was a tendency of shorter time frames between crash and arrival at the hospital during the COVID-19 era. There were 14.8% fewer transports with times of one hour or longer between the crash and facility arrival and 36.1% fewer transports that were 90 min or longer (Table S2).

#### 3.5.2. Independent Risk Factors for Mortality for Persons in or on Motorized Vehicles

Unadjusted and adjusted independent risk factors for mortality during the COVID period compared to the pre-COVID period for occupants of four-wheeled passenger vehicles, and motorized two- and three-wheeled vehicles are shown in Table 3.

In adjusted analyses, which examined variables across all five pillars of the Safe System framework, occupants of motorized two- and three-wheeled vehicles had 27% higher mortality than four-wheeled motor vehicle occupants (Table 3). Urban crash mortality was 46.7% higher. Failure to use safety equipment was associated with 44% higher mortality. Crash types and locations varied between the two study periods, with angle and sideswipe collisions and non-intersection crashes being significantly higher (Table 3). In the COVID era, more crash fatalities occurred at the scene, and thus the passengers were not transported for treatment (Table 3).

## 4. Discussion

This study identified multiple factors associated with New York State’s COVID-19-era reversal of declining mortality trends. Among the fatally injured, there was a significant degradation in safety equipment use (restraints and helmets), an increase in deaths for vulnerable vehicle types (motorized two-wheeled), an increase in deaths of those aged 30–39 years, a geographical shift toward more urban deaths, more multi-vehicle crashes, an increase in non-intersection crashes, more sideswipe and angle crashes, and more deaths on the scene that were not transported for post-crash care. Response times tended to be shorter during the pandemic, possibly due to less congested roads, but on-the-scene mortality was higher, potentially reflecting more severe injury associated with the less use of safety equipment.

While non-motorized bicycles and pedestrians contributed little to higher mortality during the examined time frame, a significant increase in motorized two-wheeled vehicle deaths contributed disproportionately—almost half of the excess increase in pandemic mortality. Mortality increases were almost seven-fold higher in unhelmeted compared to helmeted riders. This suggests a need to carefully examine ongoing federal funding policies aimed at increasing access to shared motorized micro-mobility vehicles [32], without concomitant policies that increase access to protective gear, such as helmets, which have been demonstrated to be an effective countermeasure for injury [33].

The majority of COVID-19-era pedestrian declines occurred early in the pandemic when restaurants, bars and entertainment venues were closed or operating with modified hours and to older adults who were more susceptible to adverse COVID-19 outcomes [34,35,36]. As the pandemic progressed, pedestrian deaths began to rise to pre-pandemic levels suggesting the need for further surveillance of factors associated with pedestrian deaths [37].

In unadjusted analyses, a higher proportion of drivers in the pandemic era had a history of speeding, but speeding during the pandemic era was not different. High visibility enforcement, associated with lower mortality pre-pandemic, was suspended during the pandemic and enforcement outside of this time frame was also lower [38,39,40,41]. State police traffic ticketing rebounded more quickly than local police jurisdictions. Pandemic-era enforcement was lower in incorporated urban areas where officers were charged with also addressing an increase in other criminal behaviors [39].

There are several important contributions to the literature that address travel patterns, as well as crash and traffic flow issues associated with COVID-19 lockdowns, closures and work-from-home mandates [10,11,17]. However, there is a paucity of COVID-related motor vehicle traffic injury outcomes that have a sample size adequate enough to examine the wide array of potentially contributing factors. Mortality remains higher than the pre-pandemic era. This is not fully explained by risky driving behavior. Thus, this paper is an important contribution to the literature as it uses a large sample size, an extended timeframe exactly matched with a pre-pandemic control population, and a well-defined injury outcome (death) to explore factors that were hypothesized to contribute to this continued increase in mortality. Understanding these factors is paramount to returning our roadway mortality to its pre-pandemic trajectory.

This study has limitations. Although New York State is very geographically heterogenous and experienced similar roadway mortality increases observed in other parts of the country, it was the epicenter of the pandemic and may not be representative of the U.S. [42,43]. Population-level denominators were unstable for producing rates due to pandemic shifts in travel patterns and population dynamics that left many high-rise housing complexes at one-third occupancy [27,44,45]. FARS data have well-established limitations with regard to drug data collection [25,46,47]. Lowered law enforcement roadway ticketing violations and overburdened medical examiners may have contributed to undetected COVID-19-era driver speeding and impairment [48,49,50].

## 5. Conclusions

The Safe System framework identified several previously unreported and potentially modifiable areas associated with increased pandemic era mortality. Four of the five pillars of the safe system had road safety indicators that worsened significantly during the pandemic era including: (1) urban compared to rural roadway mortality; (2) motorized two- and three-wheeled vehicles emerging as a significant contributor to pandemic era mortality increases while non-motorized bicyclists and pedestrians contributed little to the increase during the timeframe of this study; (3) lack of safety equipment use; and (4) an increase in more roadside, non-transported deaths. Lack of safety belts/helmets was associated with an increase in mortality with helmetless motorized two-wheeled rider deaths being nearly seven-fold higher than those who were helmeted. For risky behavior in motor vehicle occupants, countermeasures, such as the Click-It-Or-Ticket high visibility enforcement program, has been demonstrated to be effective at reducing mortality through addressing multiple risky driving behaviors [41]. The rapid growth of shared motorized two-wheeled vehicles has outpaced our currently available prevention approaches for safe transport on these vehicles. Further study is needed on effective approaches related to developing and enforcing rules of the road that specifically govern safety for all road users, offering road safety education, and mechanisms to provide safety equipment in the context of the rapid growth of shared motorized two- and three-wheeled vehicles. The identification of motorized two-wheeled vehicles contributing disproportionately to pandemic mortality is an emerging issue that threatens our vision to lower roadway deaths and needs further study [51]. There were troubling trends emerging regarding pedestrian mortality as the state began to re-open that deserve continued monitoring. Thus, this study both yielded important new scientific findings that can be used to focus on future prevention efforts and identified areas where additional study is needed to address emerging issues contributing to the sustained pandemic-era mortality increase.

## Figures and Tables

**Table 1:** Fatal Crashes pre-COVID and in the COVID era in New York State by road user characteristics, FARS 2018–2021.

		Pre-COVID	COVID Era	Total	
Demographic and Crash Characteristics	% Change	1 April 2018–31 December 2019	1 April 2020–31 December 2021	1 April 2018–31 December 2021	Chi-Square
		*n* (%)			X² (*p*-Value)
*n* (%)		*n* = 1725	*n* = 2010	*n* = 3735	
Road user type	16.52	1725	2010	3735	23.770 (<0.001)
Motor vehicle occupants	19.70	868 (50.3)	1039 (51.7)	1907 (51.1)	
Motorized 2-wheeled MV occupants	45.10	286 (16.6)	415 (20.6)	701 (18.8)	
Bicycle, not motorized	4.17	72 (4.2)	75 (3.7)	147 (3.9)	
Pedestrian	−6.57	487 (28.2)	455 (22.6)	942 (25.2)	
Other/unknown	116.67	12 (0.7)	26 (1.3)	38 (1.0)	
Age, collapsed (all occupants)		1153	1450	2603	11.291 (0.023)
0–12	50.0	16 (1.4)	24 (1.7)	40 (1.5)	
13–19	16.2	74 (6.4)	86 (5.9)	160 (6.1)	
20–44	41.8	521 (45.2)	739 (51.0)	1260 (48.4)	
45–64	18.7	299 (25.9)	355 (24.5)	654 (25.1)	
65+	1.2	243 (21.1)	246 (17.0)	489 (18.8)	
Safety equipment use		1641	1909	3550	86.659 (<0.001)
Unrestrained/not helmeted	52.7	364 (22.2)	556 (29.1)	920 (25.9)	
Restrained or helmeted	21.4	740 (45.1)	898 (47.0)	1638 (46.1)	
Not applicable (i.e., pedestrians)	−6.6	487 (29.7)	455 (23.8)	942 (26.5)	
Motor Vehicles (four-wheeled), Drivers					
Age		663	782	1445	18.734 (0.009)
≤19	0.0	39 (5.9)	39 (5.0)	78 (5.4)	
20–24	0.0	73 (11.0)	73 (9.3)	146 (10.1)	
25–29	41.3	63 (9.5)	89 (11.4)	152 (10.5)	
30–34	97.8	46 (6.9)	91 (11.6)	137 (9.5)	
35–39	75.0	32 (4.8)	56 (7.2)	88 (6.1)	
40–44	40.0	35 (5.3)	49 (6.3)	84 (5.8)	
45–64	5.6	198 (29.9)	209 (26.7)	407 (28.2)	
65 and over	−0.6	177 (26.7)	176 (22.5)	353 (24.4)	
Sex		664	784	1448	0.058 (0.810)
Male	18.9	512 (77.1)	609 (77.8)	1121 (77.5)	
Female	14.5	152 (22.9)	174 (22.2)	326 (22.5)	
Impaired, alcohol					0.764 (0.382)
No	21.5	488 (73.5)	593 (75.6)	1081 (74.7)	
Yes	8.5	176 (26.5)	191 (24.4)	367 (25.3)	
Restraint Status					
Driver of MV	18.1	664	784	1448	5.627 (0.018)
Unrestrained	39.8	201 (30.3)	281 (35.8)	482 (33.3)	
Restrained	5.7	407 (61.3)	430 (54.8)	837 (57.8)	
Not reported	30.4	56 (8.4)	73 (9.3)	129 (8.9)	
Motorized two-wheeled Vehicle, Drivers					
Age		271	396	667	15.433 (0.031)
<=19	33.3	9 (3.3)	12 (3.0)	21 (3.1)	
20–24	57.6	33 (12.2)	52 (13.1)	85 (12.7)	
25–29	−14.3	63 (23.2)	54 (13.6)	117 (17.5)	
30–34	106.3	32 (11.8)	66 (16.7)	98 (14.7)	
35–39	138.1	21 (7.7)	50 (12.6)	71 (10.6)	
40–44	45.0	20 (7.4)	29 (7.3)	49 (7.3)	
45–64	35.1	74 (27.3)	100 (25.3)	174 (26.1)	
65 and over	73.7	19 (7.0)	33 (8.3)	52 (7.8)	
Sex		271	397	668	0.064 (0.800)
Male	47.5	261 (96.3)	385 (97.0)	646 (96.7)	
Female	20.0	10 (3.7)	12 (3.0)	22 (3.3)	
Helmet wearing		268	397	665	9.616 (0.002)
No	235.3	17 (6.3)	57 (14.4)	74 (11.1)	
Yes	35.1	242 (90.3)	327 (82.4)	569 (85.6)	
Unknown	44.4	9 (3.4)	13 (3.3)	22 (3.3)	
Impaired, alcohol		271	397	668	1.936 (0.164)
No	57.0	193 (71.2)	303 (76.3)	496 (74.3)	
Yes	20.5	78 (28.8)	94 (23.7)	172 (25.7)	
Pedestrians					
Age		483	448	931	4.792 (0.309)
0–12	33.3	12 (2.5)	16 (3.6)	28 (3.0)	
13–19	10.0	10 (2.1)	11 (2.5)	21 (2.3)	
20–44	7.4	135 (28.0)	145 (32.4)	280 (30.1)	
45–64	−7.9	151 (31.3)	139 (31.0)	290 (31.1)	
65+	−21.7	175 (36.2)	137 (30.6)	312 (33.5)	
Sex		487	455	942	0.209 (0.648)
Male	−9.0	312 (64.1)	284 (62.4)	596 (63.3)	
Female	−2.3	175 (35.9)	171 (37.6)	346 (36.7)	
Bicyclists, Non Motorized					
Age		72	75	147	2.836 (0.586)
0–12	−66.7	3 (4.2)	1 (1.3)	4 (2.7)	
13–19	−10.0	10 (13.9)	9 (12.0)	19 (12.9)	
20–44	12.5	24 (33.3)	27 (36.0)	51 (34.7)	
45–64	33.3	21 (29.2)	28 (37.3)	49 (33.3)	
65+	−28.6	14 (19.4)	10 (13.3)	24 (16.3)	
Sex		72	75	147	1.948 (0.163)
Male	18.2	55 (76.4)	65 (86.7)	120 (81.6)	
Female	−41.2	17 (23.6)	10 (13.3)	27 (18.4)	

Note: Motor vehicle is abbreviated as MV for table readability. Unless otherwise stated, Chi square excludes unknowns.

**Table 2:** Fatal crashes pre-COVID and in the COVID era in New York State by roadway, vehicle, crash and post-crash characteristics, FARS 2018–2021.

	% Change	Pre-COVID	COVID Era	Total	
		1 April 2018–31 December 2019	1 April 2020–31 December 2021	1 April 2018–31 December 2021	Chi-Square
		*n* (%)			X² (*p*-Value)
		*n* = 1725	*n* = 2010	*n* = 3735	
Pillar II: Vehicle Characteristics					
Number of Vehicles		1725	2010	3735	10.308 (0.006)
Single	8.44	1031 (59.8)	1118 (55.6)	2149 (57.5)	
Two	22.16	564 (32.7)	689 (34.3)	1253 (33.5)	
Multiple (2+)	56.15	130 (7.5)	203 (10.1)	333 (8.9)	
Motorized Vehicle Type		1155	1461	2616	25.856 (0.007)
Car	22.43	486 (42.1)	595 (40.7)	1081 (41.3)	
SUV	19.69	193 (16.7)	231 (15.8)	424 (16.2)	
Van	18.18	44 (3.8)	52 (3.6)	96 (3.7)	
Light/Pickup Trucks	12.66	79 (6.8)	89 (6.1)	168 (6.4)	
Medium/Heavy Trucks	11.54	26 (2.3)	29 (2.0)	55 (2.1)	
Other Motorized Vehicles					
Two-Wheel Motorcycles	31.34	268 (23.2)	352 (24.1)	620 (23.7)	
Three-Wheel Motorcycles	200.00	2 (0.2)	6 (0.4)	8 (0.3)	
Moped/Motor Scooters/Minibikes	263.64	11 (0.1)	40 (2.7)	51 (1.9)	
Off-Road Motorcycles	250.00	4 (0.3)	14 (0.1)	18 (0.7)	
Other Off-Road Vehicles	42.31	26 (2.3)	37 (2.5)	63 (2.4)	
Other Vehicle Types	−72.73	11 (1.0)	3 (0.2)	14 (0.5)	
Unknown	160.00	5 (0.4)	13 (0.9)	18 (0.7)	
Collision type		1719	2008	3727	33.229 (<0.001))
Not a collision with MV in transport	7.40	1162 (67.6)	1248 (62.2)	2410 (64.7)	
Angle	24.14	261 (15.2)	324 (16.1)	585 (15.7)	
Head-on	22.65	181 (10.5)	222 (11.1)	403 (10.8)	
Rear-end	67.05	88 (5.1)	147 (7.3)	235 (6.3)	
Sideswipe	225.00	20 (1.2)	65 (3.2)	85 (2.3)	
Pillar III: Roadway Characteristics					
Urbanization		1725	2010	3735	7.670 (0.006)
Rural	0.0	494 (28.6)	494 (24.6)	988 (26.5)	
Urban	23.2	1230 (71.3)	1515 (75.4)	2745 (73.5)	
RUCC Rankings		1725	2010	3735	8.218 (0.016)
Metropolitan	20.29	1464 (84.9)	1761 (87.6)	3225 (86.3)	
Non-metropolitan, adjacent	−9.48	232 (13.4)	210 (10.4)	442 (11.8)	
Non-metropolitan, non-adjacent	34.48	29 (1.7)	39 (1.9)	68 (1.8)	
NY State Geographical Breakdown		1698	1975	3673	2.171 (0.338)
NYC	19.6	373 (22.0)	446 (22.6)	819 (22.3)	
Long Island	5.6	359 (21.1)	379 (19.2)	738 (20.1)	
Upstate	19.0	966 (56.9)	1150 (58.2)	2116 (57.6)	
Number of lanes		1140	1420	2560	9.260 (0.026)
One	13.51	37 (3.2)	42 (3.0)	79 (3.1)	
Two or more, one-way traffic	8.33	24 (2.1)	26 (1.8)	50 (2.0)	
Two or more, two-way traffic, divided	52.59	270 (23.7)	412 (29.0)	682 (26.6)	
Two or more, two-way traffic, not divided	16.19	809 (71.0)	940 (66.2)	1749 (68.3)	
Intersection type		1724	2009	3733	53.601 (<0.001)
Not an intersection	34.98	1055 (61.2)	1424 (70.9)	2479 (66.4)	
Four-way intersection	1.87	374 (21.7)	381 (19.0)	755 (20.2)	
T and Y intersections	−32.53	292 (16.9)	197 (9.8)	489 (13.1)	
Other	133.33	3 (0.2)	7 (0.3)	10 (0.3)	
Roadway Driving Conditions		1725	2010	3735	17.619 (0.002)
Clear conditions	21.68	1098 (63.7)	1336 (66.5)	2434 (65.2)	
Rain	−14.53	179 (10.4)	153 (7.6)	332 (8.9)	
Cloudy	20.88	388 (22.5)	469 (23.3)	857 (22.9)	
Sleet/Hail, Snow, Freezing Rain/Drizzle	−48.48	33 (1.9)	17 (0.8)	50 (1.3)	
Other, Fog/Smog/Smoke, Severe Crosswinds	45.45	11 (0.6)	16 (0.8)	27 (0.7)	
Unknown/Not Reported	18.75	16 (0.9)	19 (0.9)	35 (0.9)	
Traffic control devices		1154	1460	2614	12.725 (0.048)
No Controls	25.12	868 (75.2)	1086 (74.4)	1954 (74.8)	
Traffic Control Signal	33.09	139 (12.0)	185 (12.7)	324 (12.4)	
Stop Sign	−7.69	78 (6.8)	72 (4.9)	150 (5.7)	
Yield Sign	300.00	1 (0.1)	4 (0.3)	5 (0.2)	
Railway Crossing	−50.00	4 (0.3)	2 (0.1)	6 (0.2)	
Other Signs/Signals	n/a	0 (0.0)	4 (0.3)	4 (0.2)	
Unknown/Not Reported	67.19	64 (5.5)	107 (7.3)	171 (6.5)	
Lighting conditions		1725	2010	3735	7.692 (0.174)
Daylight	10.88	864 (50.1)	958 (47.7)	1822 (48.8)	
Dark, not lighted	26.64	259 (15.0)	328 (16.3)	587 (15.7)	
Dark, lighted	18.82	510 (29.6)	606 (30.1)	1116 (29.9)	
Dawn	0.00	42 (2.4)	42 (2.1)	84 (2.2)	
Dusk	37.50	48 (2.8)	66 (3.3)	114 (3.1)	
Unknown/Not Reported	400.00	2 (0.1)	10 (0.5)	12 (0.3)	
Pillar IV: Speed					
Speed Related, Motor Vehicle Drivers		664	784	1448	1.973 (0.160)
No	11.7	446 (67.2)	498 (63.5)	944 (65.2)	
Yes	31.3	217 (32.7)	285 (36.4)	502 (34.7)	
Unknown	0.0	1 (0.2)	1 (0.1)	2 (0.1)	
Speed Related, Motorized 2-wheeled MV Drivers		271	397	668	0.001 (0.981)
No	45.5	154 (56.8)	224 (56.4)	378 (56.6)	
Yes	47.9	117 (43.2)	173 (43.6)	290 (43.4)	
Pillar V: Post Crash Care					
DOA		1725	2010	3735	5.803 (0.016)
Not dead at scene or en route	9.90	1111 (64.4)	1221 (60.7)	2332 (62.4)	
Yes, DOA	29.77	608 (35.2)	789 (39.3)	1397 (37.4)	
Dead at scene	29.90	602 (99.0)	782 (99.1)	1384 (99.1)	
Dead en route	16.67	6 (1.0)	7 (0.9)	13 (0.9)	
Unknown	−100.00	6 (0.3)	0 (0.0)	6 (0.2)	
Mode of transport		1725	2010	3735	12.860 (0.012)
Not transported	30.21	609 (35.3)	793 (39.5)	1402 (37.5)	
Ambulance, ground	9.42	1083 (62.8)	1185 (59.0)	2268 (60.7)	
Ambulance, air	3.85	26 (1.5)	27 (1.3)	53 (1.4)	
Fire/police	n/a	0 (0.0)	3 (0.1)	3 (0.1)	
Other/Unknown/Not Reported	−71.43	7 (0.4)	2 (0.1)	9 (0.2)	
Time from crash to hospital arrival		564	585	1149	7.817 (0.020)
0–29 min	13.33	195 (34.6)	221 (37.8)	416 (36.2)	
30–59 min	13.64	220 (39.0)	250 (42.7)	470 (40.9)	
60+ min	−23.49	149 (26.4)	114 (19.5)	263 (22.9)	

*Notes:* MV is abbreviated as “MV” for table readability. Unless otherwise stated, Chi square excludes unknowns. For additional Safe System variables mapped onto the FARS data set, see supplemental Tables S1 and S2.

**Table 3:** Unadjusted and adjusted multivariable risk factors for mortality during the COVID-19 era compared to pre-COVID-19, FARS 2018–2021.

	Unadjusted OR (95% CI)	Adjusted Multivariable OR (95% CI)
Pillar I		
Age		
<=19	Ref	Ref
20–24	1.110 (0.692, 1.779)	1.227 (0.752, 2.003)
25–29	1.068 (0.673, 1.695)	1.101 (0.680, 1.781)
30–34	1.894 (1.174, 3.062)	1.891 (1.150, 3.116)
35–39	1.882 (1.127, 3.156)	2.025 (1.186, 3.474)
40–44	1.335 (0.791, 2.258)	1.612 (0.931, 2.798)
45–64	1.069 (0.697, 1.639)	1.267 (0.808, 1.985)
65 and over	1.004 (0.646, 1.558)	1.390 (0.869, 2.225)
Sex		
Female	Ref	Ref
Male	1.120 (0.889, 1.410)	1.035 (0.803, 1.332)
Safety Equipment		
Restrained/helmeted	Ref	Ref
Unrestrained/not helmeted	1.353 (1.123, 1.633)	1.442 (1.170, 1.780)
Pillar II		
Vehicle Type, collapsed		
Motor vehicles, four-wheeled	Ref	Ref
Motorized two- and three-wheeled vehicles	1.229 (1.020, 1.482)	1.270 (1.018, 1.585)
Collision Type		
Not a collision with MV in transport	Ref	Ref
Angle	1.016 (0.816, 1.265)	1.365 (1.033, 1.809)
Head-on	1.033 (0.808, 1.322)	1.128 (0.863, 1.476)
Rear-end	1.252 (0.911, 1.729)	1.262 (0.895, 1.791)
Sideswipe	2.848 (1.593, 5.422)	2.665 (1.464, 5.152)
Other	0.277 (0.040, 1.208)	0.276 (0.040, 1.226)
Pillar III		
Urbanization		
Rural	Ref	Ref
Urban	1.374 (1.146, 1.646)	1.467 (1.201, 1.793)
Intersection Type		
Intersection	Ref	Ref
Not an intersection	1.393 (1.149, 1.689)	1.670 (1.312, 2.128)
Pillar IV		
Speeding-related Crash		
No	Ref	Ref
Yes	1.140 (0.954, 1.362)	1.099 (0.889, 1.361)
Pillar V		
Post Crash Care		
Not transported	Ref	Ref
Transported by ground	0.859 (0.720, 1.024)	0.773 (0.636, 0.939)
Transported by air	0.838 (0.447, 1.584)	0.823 (0.433, 1.577)

OR: odds ratio with 95% confidence interval.

## Data Availability

Data are available on the Fatality Analysis Reporting System (FARS) website, https://www.nhtsa.gov/research-data/fatality-analysis-reporting-system-fars (accessed on 20 December 2024), distributed for free by the NHTSA.

## Supplementary Materials

The following supporting information can be downloaded at: https://www.mdpi.com/article/10.3390/ijerph22010061/s1, Figure S1: Total Pedestrian Mortality New York State Pre-COVID and in the COVID Era; Table S1: Fatal Crashes Pre-COVID and in the COVID Era in New York State by Road User Characteristics, FARS 2018–2021; Table S2: Fatal Crashes Pre-COVID and in the COVID Era in New York State by Roadway, Vehicle, Crash and Post-Crash Characteristics, FARS 2018–2021.
