# Supplementary material for: Using a Safe System Framework to Examine the Roadway Mortality Increase Pre-COVID-19 and in the COVID-19 Era in New York State
Source: Int J Environ Res Public Health. 2025 Jan 3;22(1):61. doi: 10.3390/ijerph22010061 (PMC11764538; doi:10.3390/ijerph22010061)
Supplement: Supplementary file 1 [file ijerph-22-00061-s001.zip › M1_PedestrianFig.Suppl (1).pptx]

## Slide 1
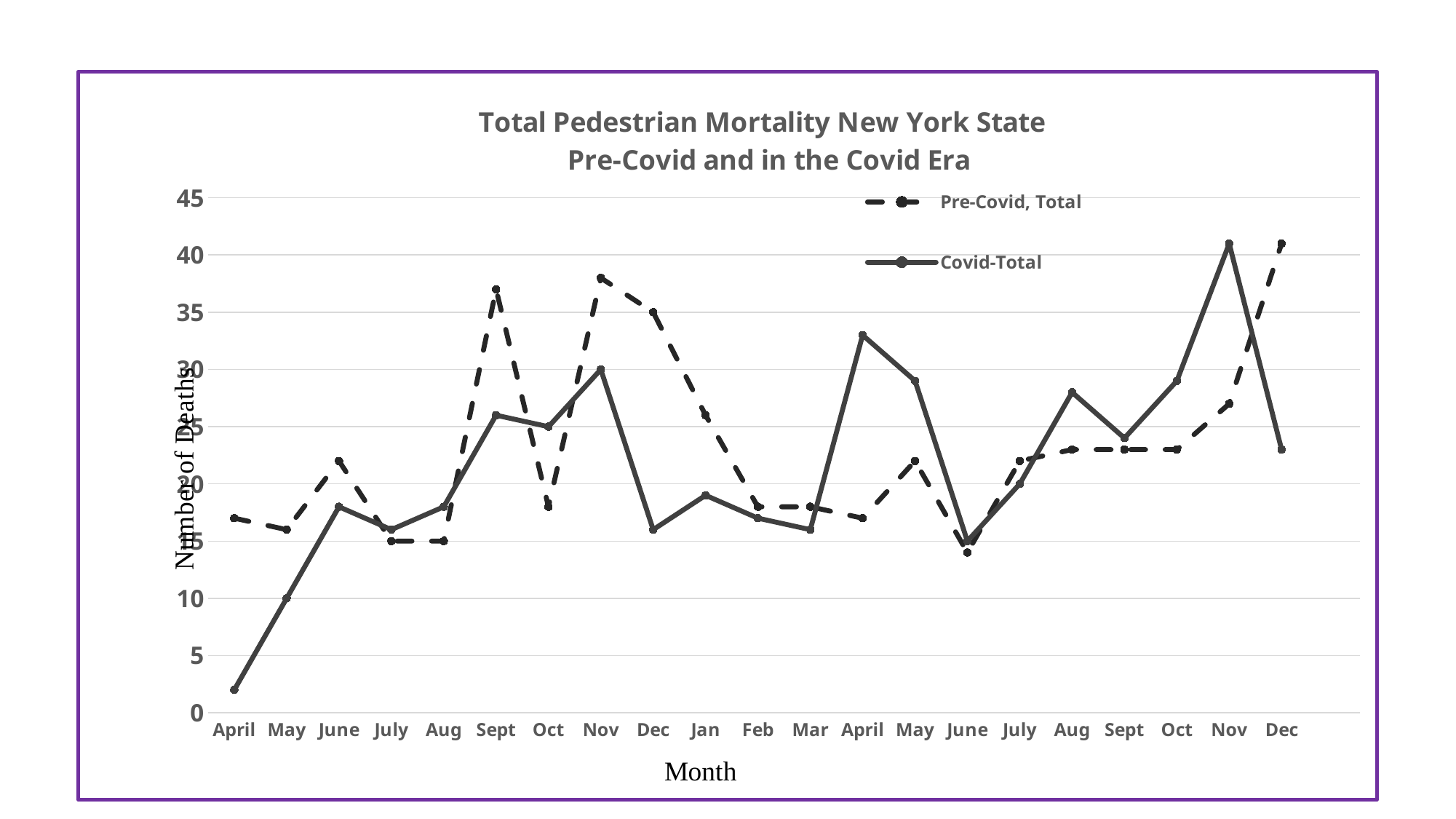

### Chart: Total Pedestrian Mortality New York State
Pre-Covid and in the Covid Era
| Category | 2 | 3 | Pre-Covid, Total | Covid-Total |
|---|---|---|---|---|
| April | None | None | 17.0 | 2.0 |
| May | None | None | 16.0 | 10.0 |
| June | None | None | 22.0 | 18.0 |
| July | None | None | 15.0 | 16.0 |
| Aug | None | None | 15.0 | 18.0 |
| Sept | None | None | 37.0 | 26.0 |
| Oct | None | None | 18.0 | 25.0 |
| Nov | None | None | 38.0 | 30.0 |
| Dec | None | None | 35.0 | 16.0 |
| Jan | None | None | 26.0 | 19.0 |
| Feb | None | None | 18.0 | 17.0 |
| Mar | None | None | 18.0 | 16.0 |
| April | None | None | 17.0 | 33.0 |
| May | None | None | 22.0 | 29.0 |
| June | None | None | 14.0 | 15.0 |
| July | None | None | 22.0 | 20.0 |
| Aug | None | None | 23.0 | 28.0 |
| Sept | None | None | 23.0 | 24.0 |
| Oct | None | None | 23.0 | 29.0 |
| Nov | None | None | 27.0 | 41.0 |
| Dec | None | None | 41.0 | 23.0 |
